# Effects of Curing Temperature on Bending Durability of Inkjet-Printed Flexible Silver Electrode

**DOI:** 10.3390/nano10122463

**Published:** 2020-12-09

**Authors:** Nam Woon Kim, Duck-Gyu Lee, Kyung-Shik Kim, Shin Hur

**Affiliations:** 1 Korea Institute of Machinery and Materials, Daejeon 34103, Korea; nwkim@kimm.re.kr (N.W.K.); educk9@kimm.re.kr (D.-G.L.); kimks@kimm.re.kr (K.-S.K.); 2 Department of Nano-Mechatronics, University of Science and Technology, Daejeon 34113, Korea

**Keywords:** flexible electrode, inkjet printing, curing temperature, bending stress, specific electrical resistance

## Abstract

Flexible electrodes should have a good mechanical durability and electrical properties under even extreme bending and deformation conditions. We fabricated such an electrode using an inkjet printing system. In addition, annealing was performed under curing temperatures of 150, 170, and 190 °C to improve the electrical resistance performance of the electrode. Scanning electron microscopy, X-ray diffraction, nanoindentation, and surface profile measurements were performed to measure and analyze the electrode characteristics and the change in the shape of the coffee ring. The bending deformation behavior of the electrode was predicted by simulations. To confirm the bending durability of the flexible electrode according to different curing temperatures, the bending deformation and electrical resistance were simultaneously tested. It was found that the electrode cured at a temperature of 170 °C could endure 20,185 bending cycles and had the best durability, which was consistent with the predicted simulation results. Moreover, the average specific resistance before the electrode was disconnected was 13.45 μΩ cm, which is similar to the conventional electrode value. These results are expected to increase the durability and life of flexible electrodes, which can be used in flexible electronic devices, sensors, and wearable devices that are subjected to significant bending deformation.

## 1. Introduction

With the development of microtechnology, the demand for flexible electrodes used in wearable and highly portable foldable devices is increasing significantly [1,2,3]. In particular, the field of flexible microelectrodes is attracting attention for many applications, including flexible displays, wearable electronic devices, and biomedical devices. To devise a flexible electrode, it is very important to minimize the changes in electrical resistance due to repetitive mechanical stresses caused due to bending, rolling, and twisting [4]. In addition, the electrode should not easily fail due to mechanical stress accumulation and should exhibit a stable and long-term electrical performance. Thus, various methods have been studied to design a precisely patterned electrode structure that satisfies these conditions. Most studies are, however, conducted using photolithography-based processes with many restrictive properties such as a high vacuum, high cleanliness, high costs, multiple process steps, and high waste liquid generation [5,6,7,8]. Therefore, it is necessary to develop a fast, simple, and inexpensive process to manufacture such a flexible electrode.

In this regard, inkjet printing is a promising new alternative technology to manufacture flexible electronic devices owing to its advantages of a simple manufacturing process, low cost, excellent large-area manufacturing capability, easy patterning, and substrate compatibility [9,10,11,12]. In general, in the inkjet printing process, nanoparticle ink that has conductive properties is deposited on a substrate by jetting, and the printed pattern is converted into a conductive electrode by curing [10]. The deposited nanoparticle ink vaporizes the solvent via curing, and the suspended particles exist in a ring-like structure at the edge contact line. This phenomenon is called the coffee ring effect or coffee stain effect [13]. Deegan et al. [14] report that when the contact line is fixed, the liquid evaporating at the edge is supplemented with an inner liquid, which carries suspended particles to the edge interface due to capillary flow. The shape of the coffee ring that appears in the inkjet printing process may cause defects in the flexible electrode, which is structurally problematic due to external mechanical stresses. Therefore, the coffee-ring effect must be minimized by controlling the inkjet printing process and curing conditions.

In the inkjet printing industry, the inks that are mainly used contain silver nanoparticles that have excellent electrical and thermal conductivities and a good chemical stability [15,16]. As nanoscale silver particles possess a low melting point, they form a conductive thin film at a relatively low temperature, which is a very important factor for the application to flexible substrates such as polymers and paper [17,18]. In general, an organic dispersant and a stabilizer are added to the silver ink formulation used in inkjet printing in order to prevent aggregation from the high surface energy of the silver nanoparticles [10,15]. Therefore, after inkjet printing, thermal curing decomposes the organic materials used to encapsulate the silver nanoparticles and aid their interaction. Therefore, post-treatment is very important in the inkjet printing process, and an optimized curing temperature is required for manufacturing high-quality, low-resistance flexible electrodes. Most of the studies on the formation of flexible electrodes have been conducted in order to improve conductive materials, manufacturing processes, annealing methods, flexibility, and electrical properties. However, few studies have been reported to simultaneously conduct experiments on the changes in the flexural durability and electrical resistance characteristics of flexible electrodes.

In this study, we fabricated silver microelectrodes using a simple inkjet printing technique and reported the characteristics of the post-treatment process at varying curing temperatures. It has been found that even at a small curing temperature range of 150–190 °C, the morphological structures of these electrodes tend to differ, thereby affecting their bending durability. In order to use the silver microelectrodes as flexible electrodes, we suggest an optimized curing temperature to impart appropriate mechanical properties and minimize the coffee ring effect. Furthermore, we confirm that the flexible inkjet-printed electrode substrate samples exhibit an excellent bending durability and specific resistance characteristics by simultaneously performing mechanical and electrical tests.

## 2. Experimental Section

### 2.1. Fabrication of Micro Silver Electrodes

**Figure 1:**
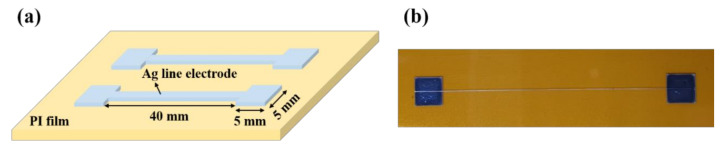
(**a**) Schematic diagram of the silver electrode designed for inkjet printing, and (**b**) Optical image of the silver electrode produced.

The silver microelectrode pattern was prepared using an inkjet printer (OmniJet 300, Uni jet Co., Gyeonggi-do, Korea) with a silver ink (DGP 40LT-15C, ANP Co., Sejong, Korea) solution on a 50-μm-thick polyimide (PI) film. As shown in Figure S1, the purchased PI film exhibited a very flat surface after Pt coating, which was confirmed by the environmental scanning electron microscope measurements (ESEM, Quanta FEG 650, FEI Co., OR, USA). The purchased silver ink solution contained 31% (wt.) of silver nanoparticles with sizes of 4–10 nm in triethylene glycol monoethyl ether (TGME) solvent (ANP Co., Sejong, Korea). In addition, the solution had a viscosity of 14.3 cps and surface tension of 34.63 mN/m. To proceed with inkjet printing, the flexible PI film was cleaned with isopropyl alcohol and flattened using vacuum. Then, inkjet printing was performed under the conditions of an ink drop volume of 1.0 pL, printing speed of 50 mm/s, frequency of 2.0 kHz, and substrate temperature of 85 °C. Silver microelectrodes were fabricated by overlapping inkjet printing 11 times with a line length of 40 mm and a line width of 45 to 50 µm between two electrical pads of 25 mm^2^ on both sides, as shown in Figure 1a,b. Once printing was complete, the silver microelectrodes were immediately transferred to a box oven that maintained the curing temperature. The printed micro silver electrodes were cured for 30 min in a box oven stably maintained at 150 °C, 170 °C, and 190 °C. In addition, ‘S’-shaped electrodes were fabricated under the same printing conditions and a curing temperature of 170 °C in order to compare the bending stability according to the electrode shape.

### 2.2. Characterization of Silver Electrode

The surface morphology of the silver electrode was observed using an environmental scanning electron microscope operating at an acceleration voltage of 30 kV. Atomic force microscopy (AFM, XE7, Park systems, Gyeonggi-do, Korea) analysis was performed to measure the surface and roughness of the micro silver electrode. The crystallinity of the silver electrode was measured with an X-ray diffractometer (XRD, Smart Lab (9 kW), Rigaku Co., TX, USA). For this, samples were scanned with Cu-Kα radiation (λ = 1.5406 Å) at a rate of 0.2°/s to observe the crystal structure. An energy dispersive X-ray analysis (EDX, Octane Pro, EDAX Co., NJ, USA) was performed to analyze the composition of the micro silver electrode at a curing temperature of 170 °C. The microelectrodes were measured according to the curing temperature using a surface profiler (alpha step IQ, KLA-Tencor, CA, USA) in order to confirm their shapes. The decomposition rate of the silver ink solution was measured in terms of the curing temperature by a high-resolution thermogravimetric analysis (TGA, TG209 F1 Libra, Netzsch, Bavaria Selb, Germany). It was conducted 1 h after the target temperature (10 °C/min) was reached using 18.5 mg of silver ink. The hardness and elastic modulus of the microelectrode were measured according to the curing temperature using a nanoindentation test system (Nanoindentation, iNano Nanoindenter, KLA-Tencor, CA, USA).

### 2.3. Tests of Bending Durability and Electrical Resistance of Micro Silver Electrode

The bending durability tests of the flexible silver electrode fabricated by inkjet printing were performed using a microforce testing system (MTS, Tytron 250, MTS systems Co., MN, USA). The test specimen was fixed to both electrode pads with a 25 mm^2^ area to expose a total length of 40 mm of the silver electrode. The bending test was performed with one jig moving a distance of 30 mm at a speed of 60 mm/s, as shown in Figure S2. During the bending durability test, electrical resistance tests were also simultaneously performed using a Source Meter (Series 2400 Source Meter, Keithley, OH, USA).

## 3. Results and Discussion

### 3.1. Morphological Characteristics of Silver Electrode

**Figure 2:**
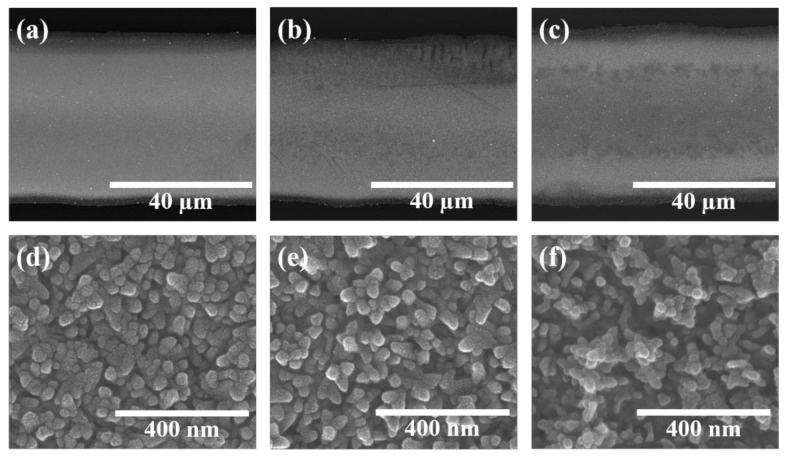
SEM images of silver electrodes fabricated at different curing temperatures; Low-magnification images at (**a**) 150 °C, (**b**) 170 °C, and (**c**) 190 °C; High-magnification images at (**d**) 150 °C, (**e**) 170 °C, and (**f**) 190 °C.

SEM images of the fabricated silver electrode cured by holding it for 30 min at a temperature of 150 °C (Figure 2a,d), 170 °C (Figure 2b,e), and 190 °C (Figure 2c,f) are shown. It was confirmed in Figure 2a–c that the printed silver nanoparticles were uniformly distributed throughout without being distorted during the curing process. The sample cured at 150 °C (shown in Figure 2d) existed as agglomerates of silver nanoparticles having sizes of 30–55 nm and was relatively close to a two-dimensional plane. The sizes of silver nanoparticle aggregates cured at 170 °C (Figure 2e) and 190 °C (Figure 2f) were 25–45 nm and 15–35 nm, respectively, and it was observed that the aggregate size decreased with an increase in the curing temperature. We performed an AFM analysis to observe the surface roughness of the silver electrodes at various curing temperatures, as shown in Figure S3. The root mean square roughness (Rq) values of the silver electrodes were 15.96, 18.05, and 20.48 nm at 150, 170, and 190 °C, respectively; this result was in accordance with the SEM measurements, which exhibited the same trend. We have elucidated the SEM images of the silver electrode before curing in Figure S4 to demonstrate how the curing process actually changes the shape of the silver electrode. It was confirmed that polygons with a size ranging from 50–110 nm lay flat on the surface of the silver electrode that did not undergo curing; consequently, it was observed that the curing process affected the size and formation of the silver electrode surface. According to the study done by Wong et al. [19], the smaller the particle size suspended in the liquid, the closer it is to the droplet contact line during the evaporation process, which results in a larger coffee ring size. It is thought that these differently-sized silver bundles influenced the shape and area of the coffee ring produced during the curing process. In addition, it was confirmed that as the curing temperature increased, the surface of the silver electrode changed to a three-dimensional structure such as a coral. It is thought that the higher the curing temperature is, the faster the evaporation of the solvent proceeds, which gradually increases the roughness. We performed XRD analyses to confirm the crystal structure of the silver electrode fabricated by the curing process, as shown in Figure S5. The XRD pattern was confirmed by the PDF card No. 01-087-0720 to be that of pure cubic structured silver. Furthermore, owing to the EDX analysis of the silver electrode performed at a curing temperature of 170 °C, it was confirmed that only pure silver was present (Figure S6).

**Figure 3:**
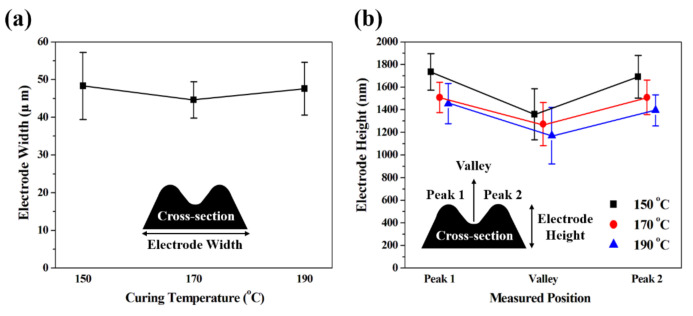
Surface profile measurement of the silver electrode fabricated at different curing temperatures; (**a**) width and (**b**) height of the electrode.

We performed a surface profile analysis to accurately determine the surface width and height of the silver electrode fabricated at different curing temperatures. A total of two samples were measured at 10 different locations (a total of 20 locations) per silver electrode prepared, and the average value is shown in Figure 3. In all of the fabricated silver electrodes, two peaks of a typical coffee ring shape were observed, whose profile is shown in Figure S7. A large coffee ring shape was already observed on the silver electrode after the inkjet printing process at 85 °C (Figure S7a). We have observed that these silver electrodes change through the curing process. Figure 3a shows the width of the silver electrode depending on the curing temperatures, and it was confirmed to be significantly reduced in comparison to that before curing (Figure S7a, 57.8 ± 2.7 μm). In addition, the electrode width and its standard deviation under the 170 °C curing condition were the smallest at 44.6 ± 4.8 μm, which means that this condition can lead to the most uniform structure of the electrode. When the curing temperatures were 150 ℃ and 190 ℃, the electrode widths and standard deviations were 48.3 ± 8.9 μm and 47.6 ± 7.0 μm, respectively, which suggested relatively thick and large profiles. This is because the printed liquid ink rapidly evaporates from the contact line during the curing process, thereby resulting in a narrow line width [13,20,21]. In addition, it is thought that the line width of the sample cured at a temperature of 190 °C partially increased due to the small size of the suspended particles [19]. Figure 3b shows the heights of the two peaks and valleys created by the coffee ring effect. As shown in the measured results, an increase in the curing temperature led to a decrease in the average electrode height. Although the same amount of silver ink was used, the height of the silver electrode was different at different curing temperatures, indicating that the high temperature increased the internal electrode density. We believe that the higher the internal density of the silver electrode is, the better it will withstand external stresses due to its rugged structure. In addition, when comparing the difference in height of the two peaks and valleys, the smallest average difference of 236.4 nm (Table S1) was found under a curing temperature of 170 °C. In terms of the electrode’s geometry, a structure with a small step difference between the heights of peaks and valleys is expected to decrease the bending stress. The inkjet-printed silver electrode confirmed that the curing temperature has a decisive influence on the formation of the coffee ring structure.

**Figure 4:**
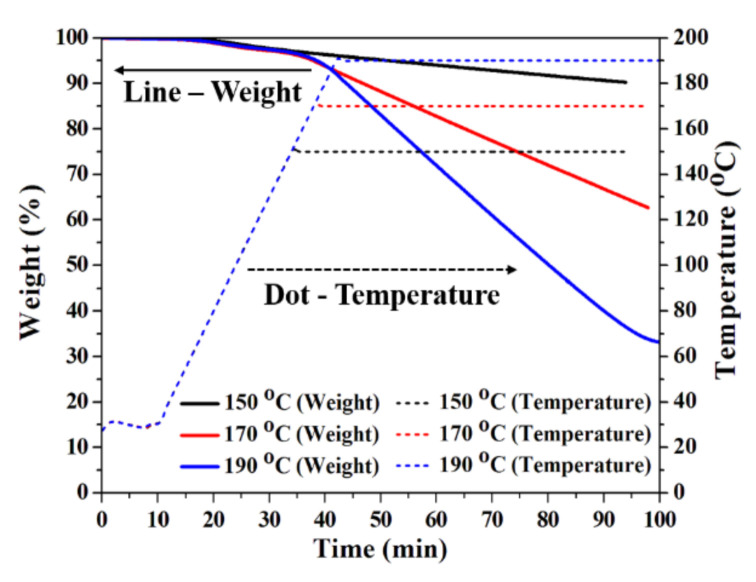
TGA analysis according to different heating times of the silver ink used to fabricate the electrode. (Straight line: weight %; Dotted line: heating temperature).

To determine the cause of the different electrode shapes according to different curing temperatures, we performed a TGA of the silver ink, and its result is shown in Figure 4. In order to perform the TGA experiment, we added 18.5 mg of silver ink containing 31% (wt.) of silver nanoparticles in a TGME solvent. The linear curve represents the weight % (left y-axis) reduced over time, and the dotted curve represents the heating temperature (right y-axis) over time. When the curing temperature was 150 °C, it was confirmed that the evaporation rate was very low (6.92%/h). In addition, it was confirmed that the evaporation rates of the solvent increased to 30.28%/h and 62.47%/h when the temperatures were 170 °C and 190 °C, respectively. We determined that the optimal solvent evaporation rate to minimize the coffee ring shape of the printed silver electrode was 5.6 mg/h. In previous studies, it was seen that the higher the applied temperature, the faster the diffusion and capillary velocities of suspended solids in the solvent, resulting in a large coffee ring shape [21,22,23]. However, the principle by which the coffee ring effect is produced should not be excluded from the surface-tension-driven flow known as the marangoni effect. Previous studies have suggested that a proper marangoni flow control can reduce or eliminate the coffee ring effect [23,24,25,26]. Based on this, we found that a curing temperature of 170 °C was optimal for minimizing the coffee ring effect due to the high temperature and marangoni effect. We conducted a bending simulation using the measured results to determine how the coffee ring structure affected the flexible electrode properties.

### 3.2. Nanoindentation Tests and Bending Simulation of the Microelectrode

**Figure 5:**
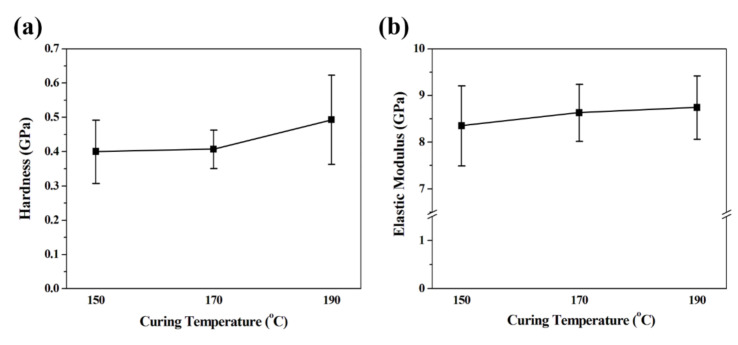
Nanoindentation results of the silver electrode fabricated according to different curing temperatures; (**a**) Hardness and (**b**) Elasticity modulus.

The silver electrode produced by inkjet printing exhibited different shapes depending on the curing temperature. We measured the hardness and elastic modulus of these samples using a nanoindentation system. During the nanoindentation, the properties were measured at 18 points per sample to calculate an average value. As shown in Figure 5, it was confirmed that both the hardness and elastic modulus increased as the curing temperature increased. When the curing temperature was at 170 °C, it was shown that there was the least error due to the silver electrode being the most uniformly formed. In addition, it can be seen that the hardness of the sample cured at 170 °C is lower than that of the 190 °C sample but that the elastic modulus, which is more important for the performance of the flexible electrode, is almost the same.

**Figure 6:**
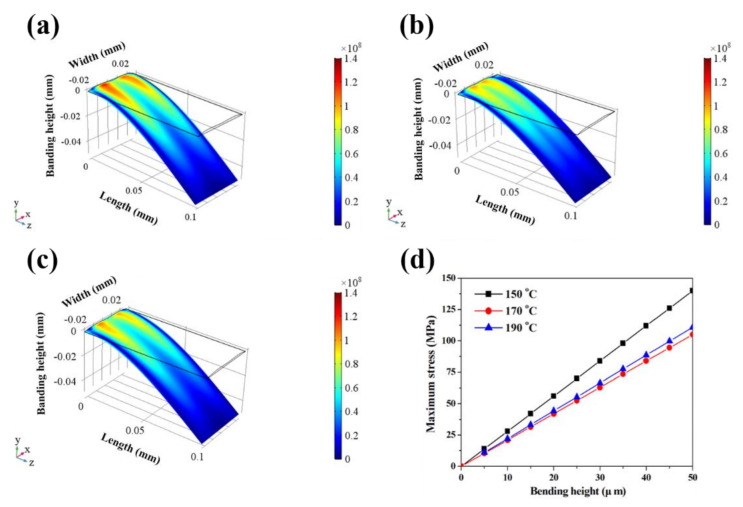
Simulation result of the silver electrode fabricated according to curing temperatures of (**a**) 150 °C, (**b**) 170 °C, (**c**) 190 °C, and the (**d**) maximum bending stress.

We performed simulations using the commercial software COMSOL in order to determine the bending stress of the electrode. In the simulation, the structure of the electrodes was modeled as a cantilever beam having a length of 100 μm. In addition, the data on the cross-sectional profiles and mechanical properties (hardness and elastic modulus) at varying curing temperatures were obtained from prior experiments and used for each electrode. Different deflections at the beam end ranging from 5 to 50 μm were given as the boundary conditions. Figure 6a–c shows the simulation results of the silver electrode according to different curing temperatures. It was shown that the area where a high bending stress occurred and led to a stress concentration was the smallest in the electrode cured at 170 °C. The largest stress concentration area was observed for a curing temperature of 150 ℃, and a high induced stress could be seen. Therefore, the silver electrode cured at a temperature of 170 °C is expected to be the most durable, which is confirmed through simulation results based on the minimum bending deformation and stress. Figure 6d shows the simulation results of the maximum stress induced by the deflection of the beam end. From these, we concluded that the inkjet-printed silver electrode must be cured at an optimal temperature of 170 °C in order to be suitable for use as a flexible electrode.

### 3.3. Bending Durability and Electrical Resistance Test of Silver Electrode

**Figure 7:**
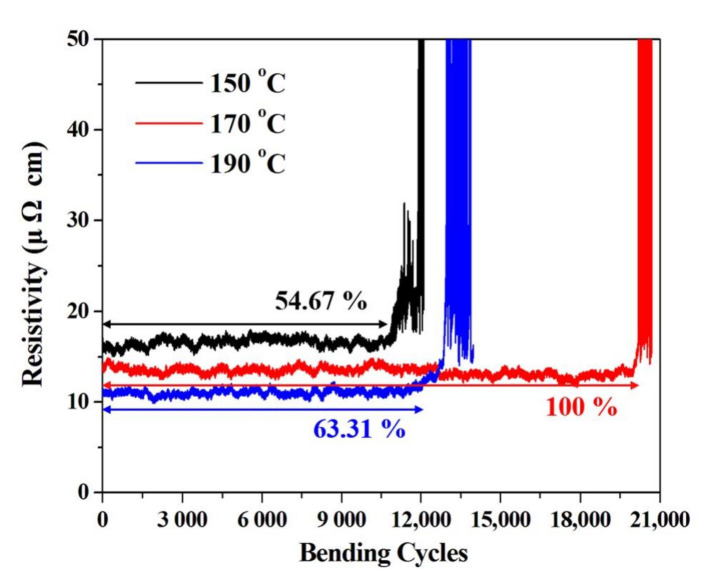
Bending durability result of the silver electrode fabricated according to the curing temperature.

**Figure 8:**
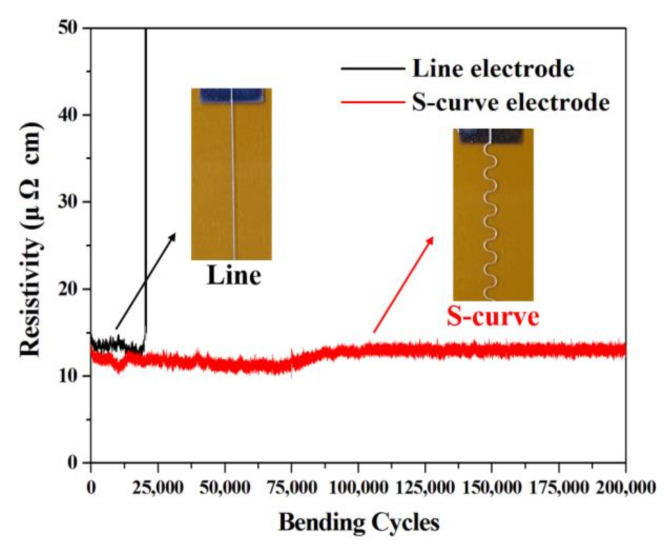
Bending durability results of the S-shaped electrode at a curing temperature of 170 °C.

We performed bending durability tests on silver electrodes cured at different temperatures using a microforce test system. To apply the maximum bending deformation to the fabricated silver electrode, one of the jigs holding the specimen was repeatedly driven from a position of 40 mm to 10 mm (Figure S2) at a speed of 60 mm/s. In addition, we performed specific resistance tests, as shown in Figure S8, in order to calculate the specific resistance, based on the resistance value simultaneously measured during the bending test and the measurement of the cross-sectional area of the surface profile and the electrode length (40 mm). Figure 7 shows the specific resistance change according to the different bending cycles of the fabricated silver electrode. The specific resistance was seen to decrease when the curing temperature increased, which was thought to be attributable to the increase in the internal density of the silver electrode and the removal of the organic dispersant contained in the silver ink. As a result of our experiment, the silver electrode maintained a constant resistivity at a curing temperature of 150 °C, but it rapidly increased after 11,035 bending cycles, which seemed to be due to the disconnection of the silver electrode. In addition, it was observed that each of the silver electrodes cured at 170 °C and 190 °C maintained a constant specific resistance and then rapidly increased after 20,185 and 12,779 bending cycles. Therefore, it was found that the bending durability was in the order of 170 °C > 190 °C > 150 °C. We consider three factors that influence the bending durability: (1) the small step structure of the peaks and valleys obtained by reducing the coffee ring effect, (2) the uniform film size and shape produced by the appropriate solvent evaporation rate, and (3) the hardness and elastic modulus of the flexible silver film at different curing temperatures. From this point of view, the sample cured at 170 °C showed the lowest difference in height between the peaks and valleys, resulted in the most uniform thickness, and showed an excellent hardness and modulus of elasticity, which was consistent with the bending durability results. As predicted in the simulation, it was confirmed that the bending durability of the silver electrode was the best when it was cured at 170 °C. We succeeded in fabricating a flexible silver electrode by obtaining the optimal curing temperature of the printed silver electrode. However, the silver electrode ruptured after 20,185 bending cycles, after which the electrical resistance increased rapidly. To improve this, we fabricated flexible S-shaped electrodes with 1.0 mm radius curvature using an inkjet printer and performed similar bending durability tests. In general, S-shaped patterns are known to be stronger than linear patterns under tensile stress [27,28,29,30,31,32]. As shown in Figure 8, the flexible S-shaped electrode showed a consistent bending durability even after 200,000 bending cycles. When the line electrode (x direction) is bent, the total bending tension acts in the x direction. Therefore, stress that causes cracking is accumulated in the y direction in the line electrode. This separates the line electrode and reduces the bending durability [33,34]. However, in the S-shaped pattern, the bending tension is divided by the x and y directions during bending [27,30,31]. This result means that the S-shaped electrode receives relatively less bending stress than the line electrode. For this reason, it is believed that the S-shaped electrode showed a superior bending durability than the line electrode. We confirmed the significance of the curing temperature of the inkjet-printed electrode and the fact that the bending durability of the electrode could be improved by simply changing the pattern shape. The flexible silver microelectrode fabricated in this study will be suitable for use in a variety of flexible devices with improved service lives.

## 4. Conclusions

We fabricated silver electrodes using inkjet printing technology and optimized the curing temperature required for their use as flexible electrodes. When a curing temperature of 170 °C was used, the coffee ring effect of the silver electrode was seen to be minimized, and the width and height of the silver electrode were the most uniform. We conducted simulations of the bending stress using results analyzed according to different curing temperatures. It was confirmed that a minimized coffee ring structure contributed to the lowest bending stress. We also evaluated the bending durability of the fabricated silver electrode, and the optimized sample showed a superior durability performance, higher by approximately 46.7% (7406 bending cycles) when compared to those of other conditions. In addition, we investigated the bending durability of the electrode pattern under the optimized curing temperature using an S-shaped pattern, which showed a 10-fold better bending durability than that of the linear one. We expect that the flexible silver electrode with excellent durability, fabricated in this study, can be used as a micro component in various applications.

## Supplementary Materials

The following are available online at https://www.mdpi.com/2079-4991/10/12/2463/s1, Figure S1. SEM image of Pt-coated PI film used as a substrate. Figure S2. Optical photo showing the experimental setup for measuring the bending durability and specific electrical resistance of silver electrodes fabricated according to the curing temperature; (a) before bending, (b) after bending. Figure S3. AFM images of silver electrodes fabricated at various curing temperatures; (a) 150 °C, (b) 170 °C, (c) 190 °C. Figure S4. SEM images of silver electrode without the curing process; (a) Low-magnification, (b) High-magnification. Figure S5. XRD patterns of silver electrodes fabricated at various curing temperatures. Figure S6. EDX spectrum of the silver electrode at a 170 °C curing temperature. Figure S7. Surface profile results close to the average value of silver electrodes fabricated at varying curing temperatures; (a) without curing process, (b) 150 °C, (c) 170 °C, (d) 190 °C. Figure S8. Specific resistance of the silver electrode fabricated at varying curing temperatures. Table S1. The mean value and standard deviation of the profile analysis result of the silver electrode fabricated at varying curing temperatures.
