# Supplementary material for: Effects of Curing Temperature on Bending Durability of Inkjet-Printed Flexible Silver Electrode
Source: Nanomaterials (Basel). 2020 Dec 9;10(12):2463. doi: 10.3390/nano10122463 (PMC7763182; doi:10.3390/nano10122463)
Supplement: Supplementary file 1 [file nanomaterials-10-02463-s001.pdf]

Supplementary Materials

Effects of Curing Temperature on Bending Durability of Inkjet-Printed Flexible Silver Electrode

Nam Woon Kim [1], Duck-Gyu Lee [1], Kyung-Shik Kim [1] and Shin Hur [1,2,*]

[1] Korea Institute of Machinery and Materials, Daejeon 34103, Korea; nwkim@kimm.re.kr (N.W.K.); educk9@kimm.re.kr (D.-G.L.); kimks@kimm.re.kr (K.-S.K.)

[2] Department of Nano-Mechatronics, University of Science and Technology, Daejeon 34113, Korea

* Correspondence: shur@kimm.re.kr; Tel.: +82-42-868-7886

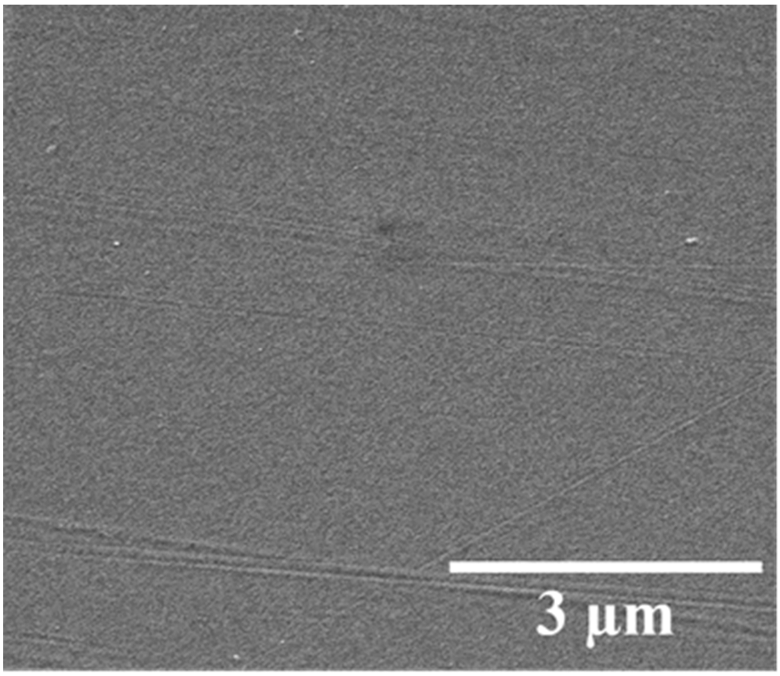

Figure S1. SEM image of Pt-coated PI film used as a substrate.

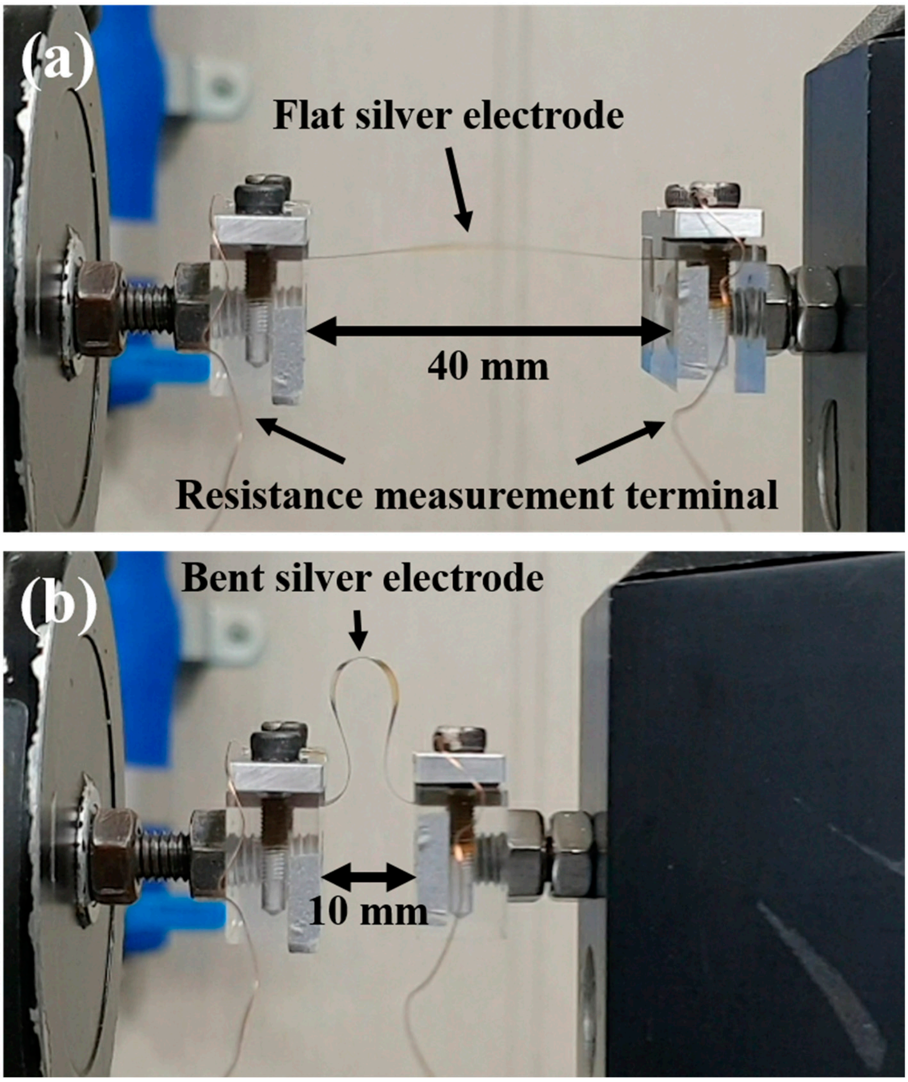

Figure S2. Optical photo showing the experimental setup for measuring the bending durability and specific electrical resistance of silver electrodes fabricated according to the curing temperature; (a) before bending, (b) after bending.

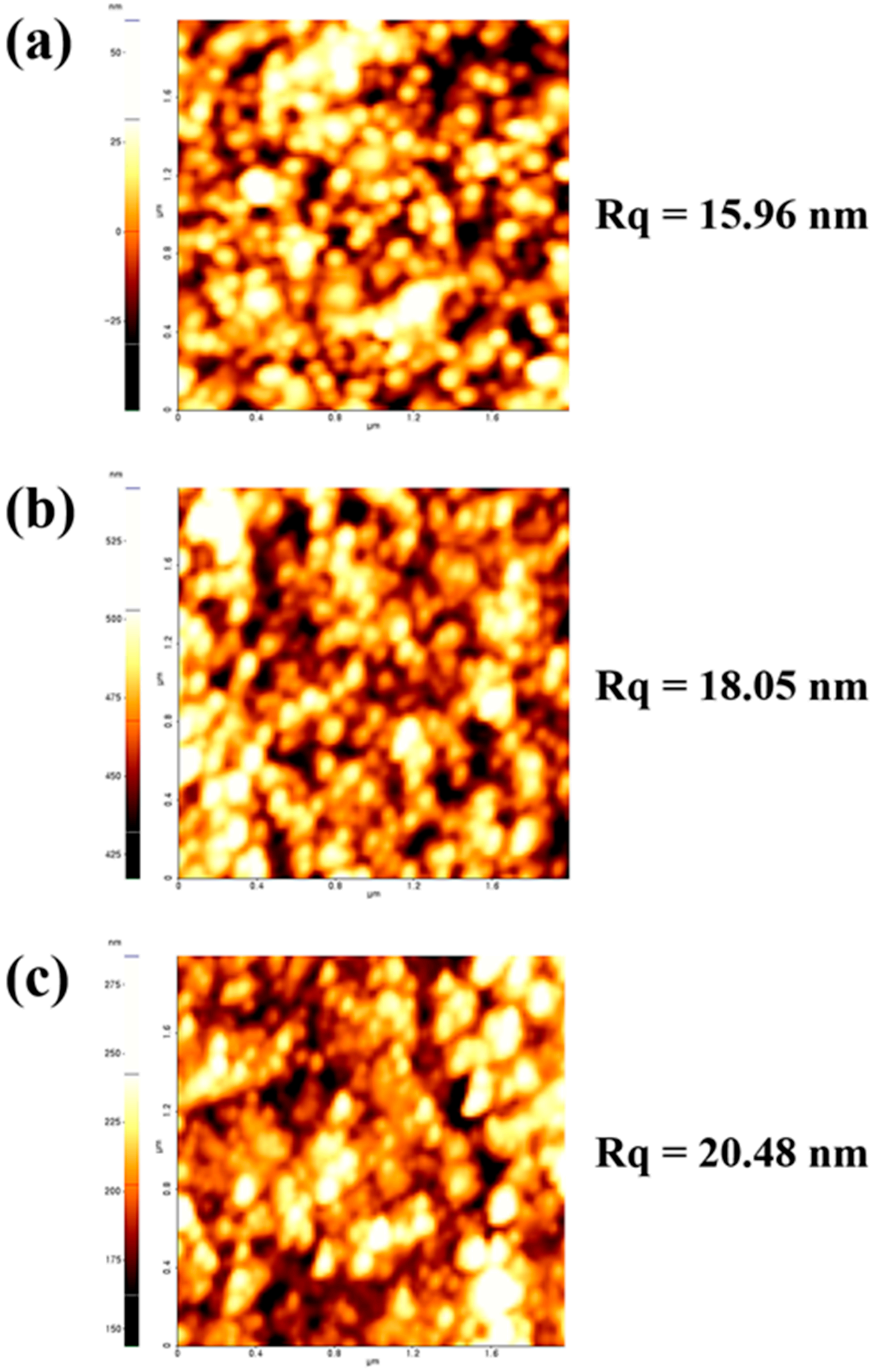

Figure S3. AFM images of silver electrodes fabricated at various curing temperatures; (a) 150 °C, (b) 170 °C, (c) 190 °C.

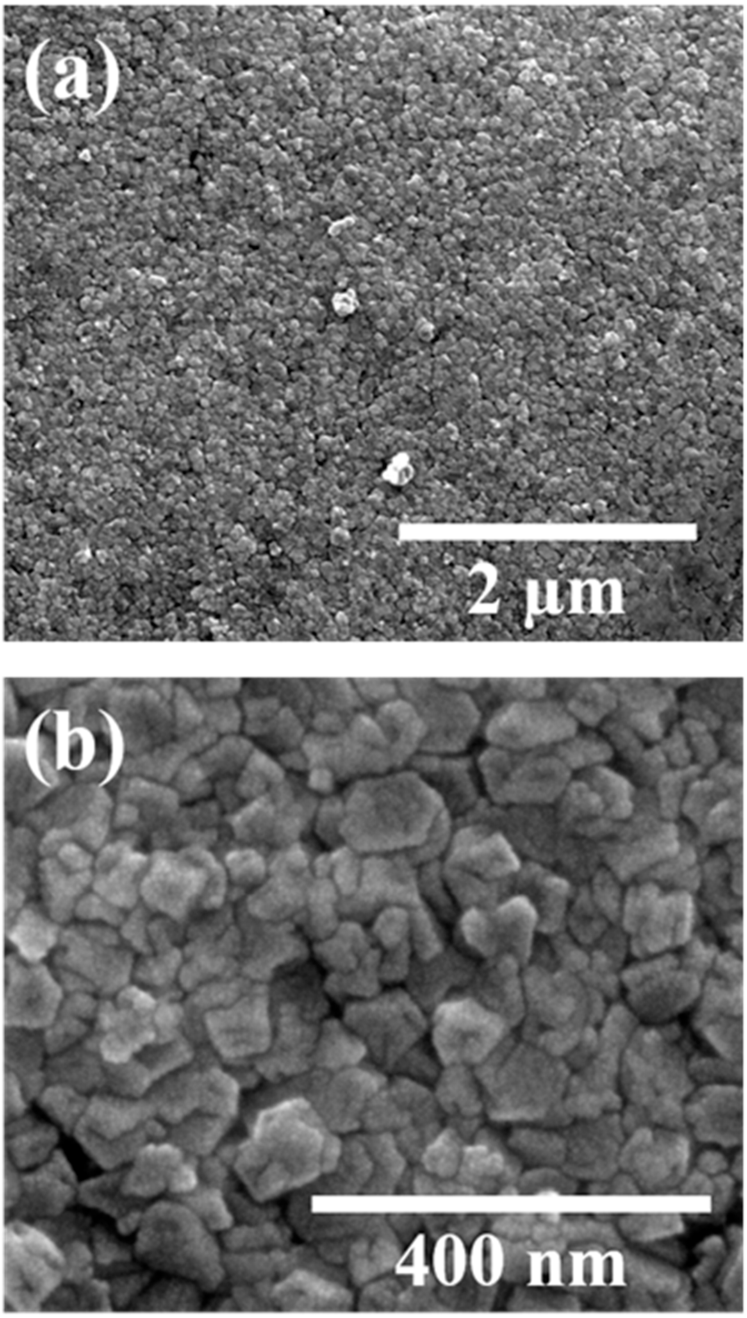

Figure S4. SEM images of silver electrode without curing process; (a) Low-magnification, (b) High-magnification.

nanomaterials

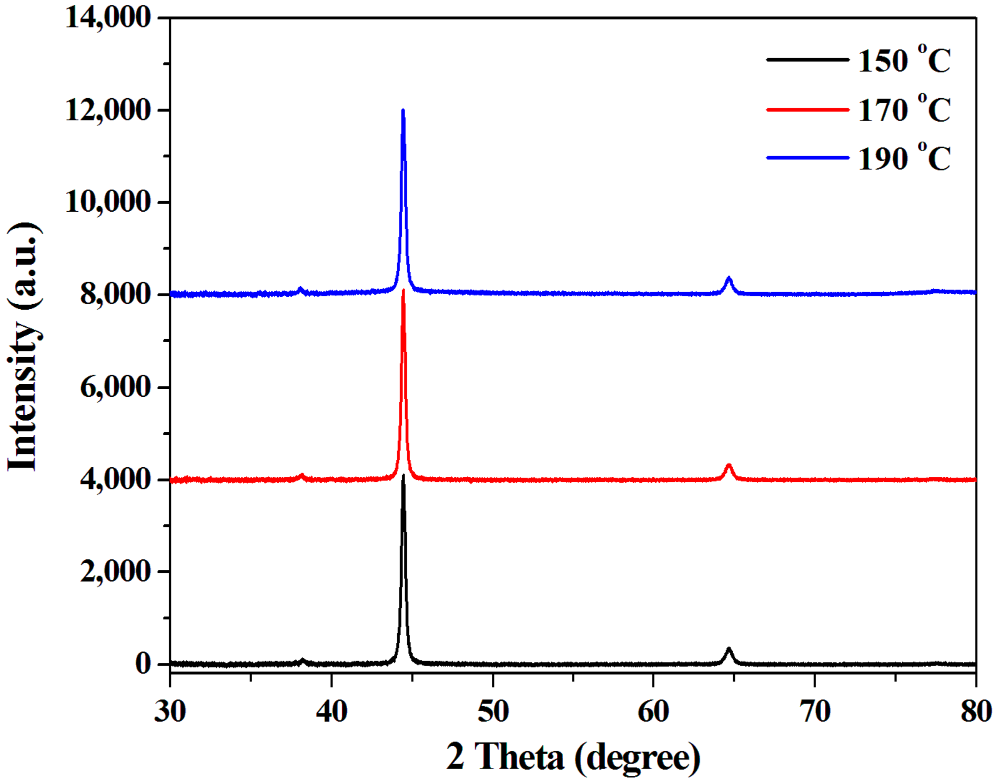

Figure S5. XRD patterns of silver electrodes fabricated at various curing temperatures.

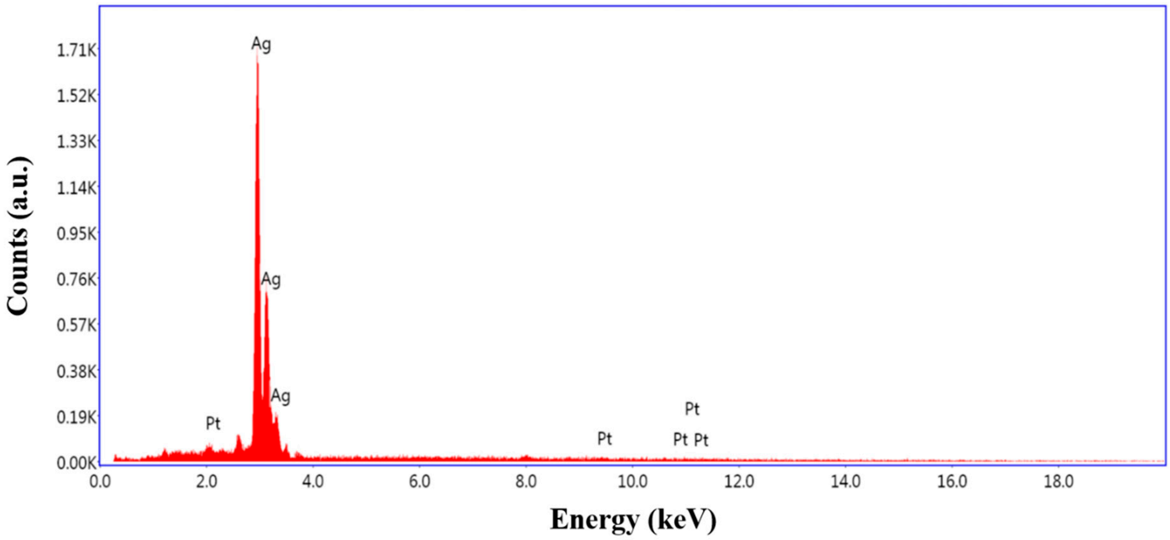

Figure S6. EDX spectrum of silver electrode at 170 °C curing temperature.

nanomaterials

MDPI

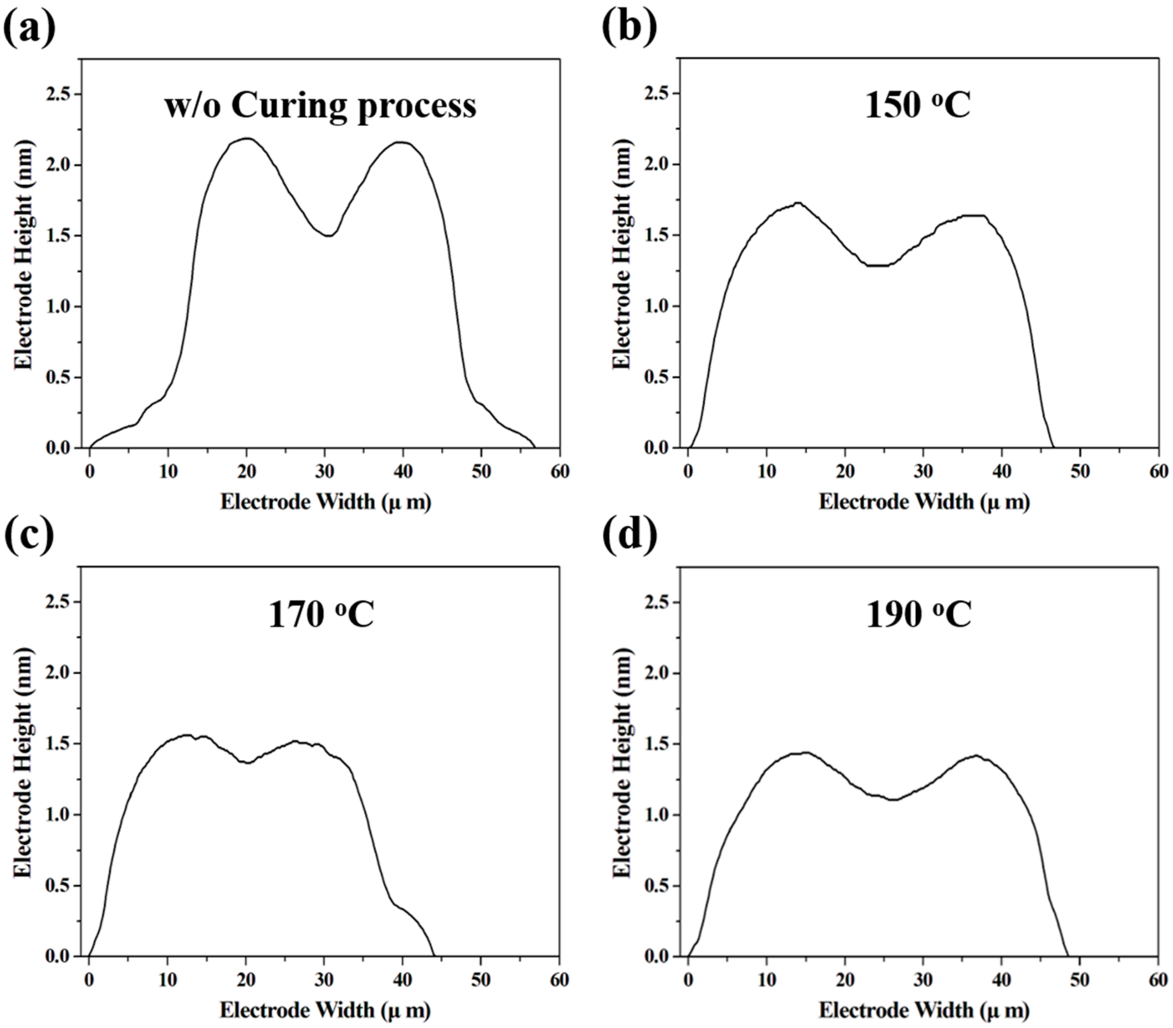

Figure S7. Surface profiles results close to the average value of silver electrodes fabricated at varying curing temperature; (a) without curing process, (b) 150 °C, (c) 170 °C, (d) 190 °C.

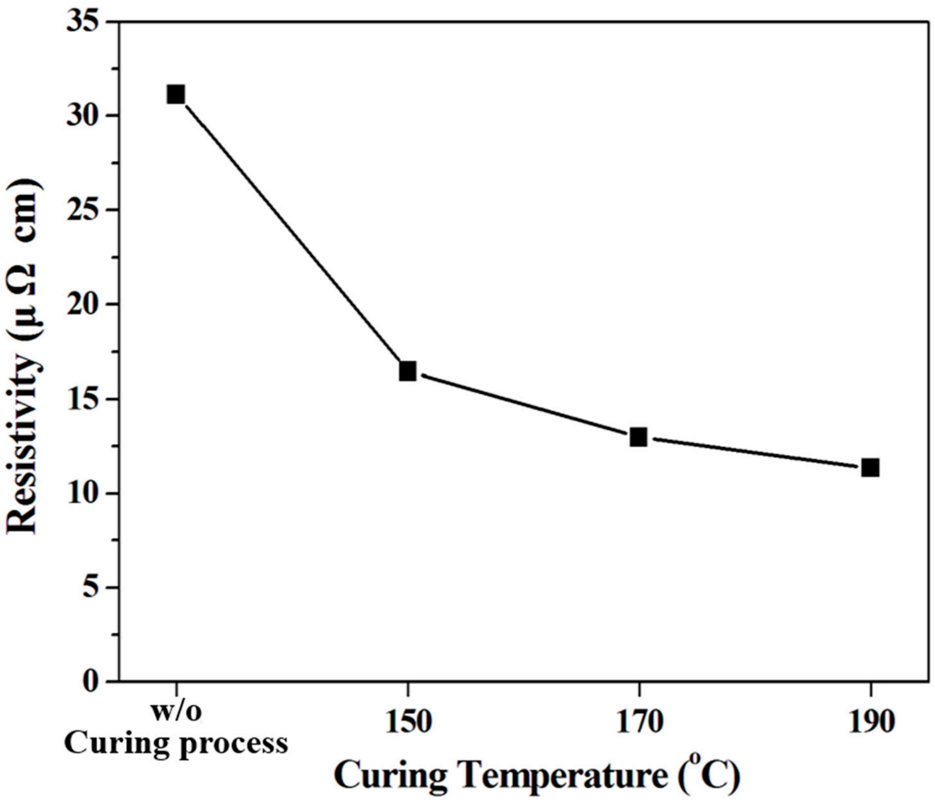

Figure S8. Specific resistance of silver electrode fabricated at varying curing temperature.

Table S1. The mean value and standard deviation of the profile analysis result of the silver electrode fabricated at varying curing temperature.

Sample	Electrode Width (μm)	Electrode Height (nm)				
		Peak 1	Valley	Peak 2	Peak 1 − Valley	Peak 2 − Valley
w/o curing process	57.8 (±2.7)	2146.2 (±65.3)	1504.2 (±150.0)	2153.2 (±53.7)	642.0	649.0
150 °C	48.3 (±8.9)	1734.7 (±162.6)	1359.9 (±226.3)	1692.0 (±189.2)	374.8	332.1
170 °C	44.6 (±4.8)	1509.3 (±134.1)	1273.2 (±190.7)	1509.8(±152.8)	236.1	236.7
190 °C	47.6 (±7.0)	1454.2 (±179.2)	1171.2 (±249.1)	1394.5 (±138.0)	283.0	223.4
